# Neocortical morphometry in Huntington's disease: Indication of the coexistence of abnormal neurodevelopmental and neurodegenerative processes

**DOI:** 10.1016/j.nicl.2020.102211

**Published:** 2020-02-13

**Authors:** Jean-Francois Mangin, Denis Rivière, Edouard Duchesnay, Yann Cointepas, Véronique Gaura, Christophe Verny, Philippe Damier, Pierre Krystkowiak, Anne-Catherine Bachoud-Lévi, Philippe Hantraye, Philippe Remy, Gwenaëlle Douaud

**Affiliations:** a Université Paris-Saclay, CEA, CNRS, Baobab, Neurospin, Gif-sur-Yvette, France; b Commissariat à l'Energie Atomique et aux Energies Alternatives (CEA), Département des Sciences du Vivant (DSV), Institut d'Imagerie Biomédicale (I2BM), MIRCen, France; c Centre national de référence des maladies neurogénétiques, Service de neurologie, CHU, 49000 Angers, France, UMR CNRS 6214 – INSERM U1083, France; d CHU Nantes, INSERM, CIC 0004, France; e Neurologie, CHU Amiens-Picardie, France; f AP-HP, Hôpital Henri Mondor, Centre de Référence-Maladie de Huntington, France; g MIRCen, Institut d'Imagerie Biomédicale, Direction de la Recherche Fondamentale, Commissariat à l'Energie Atomique et aux Energies Alternatives, France; h Functional Magnetic Resonance Imaging of the Brain (FMRIB) Centre, Wellcome Centre for Integrative Neuroimaging, Nuffield Department of Clinical Neurosciences, University of Oxford, United Kingdom

**Keywords:** Huntington's disease, MRI, Cortical morphometry, Sylvian fissure, Neurodevelopment, Asymmetry

## Abstract

Huntington's disease (HD) is an inherited, autosomal dominant disorder that is characteristically thought of as a degenerative disorder. Despite cellular and molecular grounds suggesting HD could also impact normal development, there has been scarce systems-level data obtained from *in vivo* human studies supporting this hypothesis. Sulcus-specific morphometry analysis may help disentangle the contribution of coexisting neurodegenerative and neurodevelopmental processes, but such an approach has never been used in HD. Here, we investigated cortical sulcal depth, related to degenerative process, as well as cortical sulcal length, related to developmental process, in early-stage HD and age-matched healthy controls. This morphometric analysis revealed significant differences in the HD participants compared with the healthy controls bilaterally in the central and intra-parietal sulcus, but also in the left intermediate frontal sulcus and calcarine fissure. As the primary visual cortex is not connected to the striatum, the latter result adds to the increasing *in vivo* evidence for primary cortical degeneration in HD. Those sulcal measures that differed between HD and healthy populations were mainly atrophy-related, showing shallower sulci in HD. Conversely, the sulcal morphometry also revealed a crucial difference in the imprint of the Sylvian fissure that could not be related to loss of grey matter volume: an absence of asymmetry in the length of this fissure in HD. Strong asymmetry in that cortical region is typically observed in healthy development. As the formation of the Sylvian fissure appears early *in utero*, and marked asymmetry is specifically found in this area of the neocortex in newborns, this novel finding likely indicates the foetal timing of a disease-specific, genetic interplay with neurodevelopment.

## Highlights

• We found shallower central, intraparietal and left intermediate frontal sulci in HD.
• Shallow calcarine fissure is further evidence of primary cortical degeneration in HD.
• Healthy subjects show strong asymmetry in length of posterior Sylvian fissure (pSF).
• Absence of pSF asymmetry in HD indicates genetic interplay with neurodevelopment.

## 1 Introduction

Cortical sulcal analysis has for long solely relied on the empirical description of the cortical foldings investigated post mortem (Dareste, 1852; Broca, 1878). At the antenatal stage, two fundamental steps, now thought to be common to the higher order mammals, had been observed: first, the operculisation of the insula at 6 months, followed by a progressive gyrification allowing for the neocortical surface to increase and become more complex in the last three months of development. These historical observations prefigured a theory that poses the stability of these sulcal “roots” across individuals, something which was further observed *in vivo* in newborns (Regis et al., 2005; Dubois et al., 2008a; Dubois et al., 2008b). Furthermore, it has been known since the beginning of the 19th century that various developmental abnormalities leading to cortical sulci malformation are associated with sensorimotor, cognitive or behavioural disorders (Bruce, 1889; Cameron, 1907). Investigating sulcal morphometry might thus capture abnormalities emerging during neocortical development, either chronologically coinciding to the formation of sulcal roots, or later during cortical maturation process.

Huntington's disease (HD) is a fatal autosomal dominant, neurodegenerative disorder resulting from an expansion of a CAG repeat within the IT15 gene on chromosome 4. While the striatum is the most atrophied structure in HD, there is evidence that the cortical atrophy is more widespread than previously thought based on *post mortem* observations, this loss of volume sometimes appearing even before the onset of symptoms (Rosas et al., 2002; Thieben et al., 2002; Rosas et al., 2005; Douaud et al., 2006; Rosas et al., 2008). Importantly, two recent *in vivo* studies of global anthropometric measures in asymptomatic subjects carrying the mutated gene also point at a developmental aspect in HD (Nopoulos et al., 2011; Lee et al., 2012). These are, to our knowledge, the only human studies showing results supporting the thought-provoking idea that degeneration in some disorders of possible genetic aetiology, including HD and Alzheimer's disease, might be the consequence of abnormal development, with certain populations of neuronal cells made more vulnerable to late life stressors (Mehler and Gokhan, 2000; Molero et al., 2009; Marder and Mehler, 2012).

Here, we carried out for the first time in HD a sulcal morphometry analysis using a tool that automatically reconstructs and labels sulci from T1-weighted images (Riviere et al., 2002; Mangin et al., 2004). This approach has revealed for instance significant phylogenetic differences in a language-related sulcal area (Leroy et al., 2015), or alterations in sulcal shape in ageing (Kochunov et al., 2005) – with, for instance, a reduced sulcal depth related to adjacent gyral atrophy – as well as in mild cognitive impairment and Alzheimer's disease (Reiner et al., 2012; Hamelin et al., 2015). Furthermore, differences in sulcal length have been recently consistently related to (abnormal) developmental processes (Auzias et al., 2014; Cachia et al., 2014; Muellner et al., 2015).

We thus expected that sulcal morphometry analysis might reveal evidence for coexisting abnormal degenerative and developmental processes, in line with the duality, observed for the mutant protein, of both gain-of-function and loss-of-function (effects which are in turn thought to play a distinct role in brain degeneration and abnormal development respectively) (Marder and Mehler, 2012). As this exploratory, yet region-of-interest based approach provides information on the shape of sulci complementary to information obtained with voxelwise techniques, we anticipated that it should in particular detect subtle abnormalities not identified using an approach such as VBM (Mangin et al., 2004; Douaud et al., 2006) and that it might, crucially, reveal novel abnormalities related to altered neurodevelopment in HD.

## 2 Methods

This study was part of the MIG-HD project (Multicentric Intracerebral Grafting in Huntington's Disease) and was approved by the ethics committee of Henri Mondor Hospital in Créteil. All subjects gave written informed consent.

### 2.1 Participants

Twenty-three HD patients (14 males, 9 females, 2 left-handed, aged 42 ± 8 years, range 25–54) were included from four different hospitals (Nantes, Angers, Lille and Créteil). All were scanned using the same scanner, in the same imaging centre in Orsay. To meet inclusion criteria, all had genetically proven HD, with an abnormal number of CAG repeats ranging from 40 to 57 (46 ± 4). None had juvenile HD. They all had clinical symptoms for at least 1 year and 15 were at stage I of the disease according to their total functional capacity score (TFC ≥ 11) (Shoulson and Fahn, 1979), i.e., they were autonomous and could function fully both at work and at home (on average 10.9 ± 1.4, range 8–13). 18 healthy controls (HC, 14 males, 4 females, 2 left-handed) matched for age (41 ± 8 years) to the HD patients underwent the same imaging protocol. Each HD patient was examined using the Unified Huntington's Disease Rating Scale (UHDRS, 1996) in each hospital and the scores for each subscale (motor, behavioural, functional and neuropsychological) were collected (Table 1).

**Table 1:** **Clinical variables for the HD participants.**

Clinical Variable	Mean±std	Range
CAG repeat	46±4	40–57
Total Functional Capacity	11±1	8–13
Disease Burden	409±73	239–538
Motor UHDRS	35±14	16–61
Behavioural UHDRS	12±10	0–36
Functional Assessment	27±2	25–31
Independence Scale	88±9	70–100
Verbal Fluency (P, R, V) – 1min	27±10	7–43
Verbal Fluency (P, R, V) – 2min	37±13	14–62
Digit Symbol	26±9	14–48
Stroop (Words)	63±21	29–103
Stroop (Colour)	46±15	24–76
Stroop (Interference)	26±9	10–43

### 2.2 Data acquisition

Whole-brain anatomical MRI was acquired in all 41 participants with a 1.5 T Signa imager (General Electric Healthcare, Milwaukee, WI) with a standard 3D T1-weighted inversion recovery fast spoiled gradient recalled (IR-FSPGR) sequence with the following parameters: axial orientation, matrix 256 × 256, 124 slice locations, 0.9375 × 0.9375 mm^2^ in-plane resolution, slice thickness 1.2 mm, TI/TE/TR (inversion/echo/repetition time) 600/2/10.2 ms, flip angle (α) 10°, read bandwidth (RBW) 12.5 kHz.

### 2.3 Image processing

Here is a brief description of the main steps implemented in BrainVISA for the reconstruction of the sulci http://brainvisa.info (Mangin et al., 2004).

First, T1-weighted images were corrected for inhomogeneities and a brain mask (grey matter GM and white matter WM) was created for each image, based on the analysis of the histogram and a morphological opening, before being segmented into left and right hemispheres, as well as cerebellum. Next, the complement of the white matter, defined as the space between the brain envelope (identified using a morphological closing) and the GM/WM boundary (identified from the intensities of the two tissues), was skeletonised to create a 3D print of each sulcus. We thus obtained the 3D reconstruction of sulci for each of the 23 HD patients and 18 healthy controls.

Various sulcal features can then be analysed, but here we focused on two that are easily interpretable: depth and length of the sulcus. Decrease of depth of sulci has been consistently reported in case of neurodegeneration (with healthy ageing and Alzheimer's disease), as the sulci become more shallow as adjacent gyri degenerate (Kochunov et al., 2005; Reiner et al., 2012; Hamelin et al., 2015). In contrast, differences in length of the sulci are thought to relate to abnormal developmental processes (Auzias et al., 2014; Cachia et al., 2014; Muellner et al., 2015).

As there is a substantial inter-subject variability in the shape and location of the sulci, making a non-linear warping to standard space approach not appropriate, the strategy here was to use the automatic recognition of the sulci based on supervised learning from a database created by neurosurgeons and using neural networks (Riviere et al., 2002). This process relies on energy minimisation and in this specific case three successive annealings, where we selected the one which minimised best the system's energy.

To create an additional variable, we manually delineated the striatal regions on each axial plane of each individual T1-weighted scan, after all the images were rigidly reoriented so that the anterior and posterior commissures were located in the same axial plane (Douaud et al., 2006). The accuracy of delineation was further checked in both sagittal and coronal planes, and each striatal region was reconstructed in 3D to control for the shape of each volume created. We then calculated the asymmetry index of the striatal regions to further correlate with possible results showing a marked unilateral effect.

### 2.4 Statistical analysis

We carried out an ANCOVA to compare sulci between the two populations, with diagnosis, age, and age by diagnosis interaction as covariates to make the results easily comparable with a previous voxel-based study in this population (18 out of 23 HD patients in common) (Douaud et al., 2006). Results were considered significant for *P* < 0.05 (two-tailed), corrected for false discovery rate (FDR) across all sulci (*n* = 57).

We additionally checked that our sulcal results held when: 1. adding sex and handedness as additional covariates, 2. normalising for intracranial volume by calculating the residuals for depth and length after the linear contribution of the intracranial volume to the power 1/3 was removed (Sanfilipo et al., 2004).

We further ensured that our results showing differences in the length of the sulci – presumably of developmental nature – were in fact not associated with disease burden ((nCAG-35.5) × age) or disease stage (TFC). To this effect, we calculated the correlation coefficient within the HD group between these two clinical measures and our imaging measures of length showing significant group differences.

In addition, we investigated within the HD group whether any of our significant findings might be correlated *a posteriori* with their behavioural and clinical scores (Table 1) using Pearson correlation (with and without age added as a covariate of no interest), as well as with their striatal volumetric asymmetry for asymmetric finding. To account for multicollinearity of these scores, we reduced the set of clinical scores to those that did not share more than 50% of explained variance.

Normality of the data was tested in R for every statistical analysis (using the Datamind software of BrainVISA) (Duchesnay et al., 2007).

## 3 Results

**Fig. 1:**
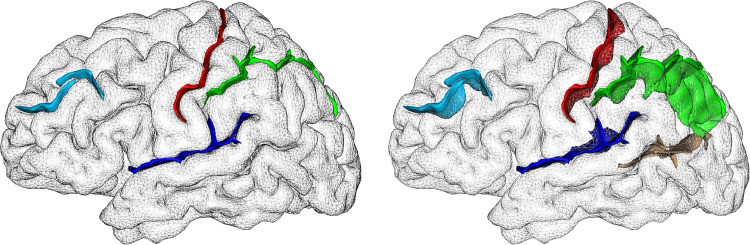
**Visual representation of some of the sulci found the most different between healthy and HD participants (5 of 7).** We show the sulci in the left hemisphere of one randomly selected healthy control: left, opaque cortex; right, partially transparent cortex to visualise the 3D conformation of the sulci, and those on the medial surface. The central sulcus appears in red, the intra-parietal sulcus in green, the posterior lateral fissure in dark blue, the intermediate frontal sulcus in light blue, and by transparency, the calcarine fissure in brown. While the results in the central sulcus and intra-parietal sulcus were bilateral, differences in the posterior lateral fissure, intermediate frontal sulcus and calcarine fissure were left-lateralised.

Several sulci were significantly abnormal in the HD patients (Table 2, Fig. 1, FDR-corrected). While most of the measures that differed between the two populations were atrophy-related, showing shallower sulci in HD (8 out of 9 of the significant findings), one measure could not be related to loss of grey matter volume seen in this neurodegenerative disorder: the length of the left posterior Sylvian fissure. These results held when adding sex and handedness to the statistical model, as well as after normalising for intracranial volume

**Table 2:** **All significant (FDR-corrected) sulcal differences between HD (*****n*** **=** **23) and healthy controls (HC,*****n*** **=** **18).**

Sulci	Side	Feature	HC Mean±std	HD Mean±std	*P*-value	*P*-value*(Sex+Handedness)	*P*-value⁎⁎(ICV)
Central Sulcus	L	Depth	22.6 ± 1.3	21.2 ± 1.1	4.0 × 10^−4^	1.9 × 10^−3^	1.8 × 10^−2^
	R	Depth	23.0 ± 1.2	21.0 ± 1.3	4.6 × 10^−6^	3.0 × 10^−5^	2.1 × 10^−3^
Intra-parietal sulcus	L	Depth	25.1 ± 1.5	23.0 ± 1.8	2.9 × 10^−5^	1.3 × 10^−4^	5.2 × 10^−4^
	R	Depth	24.4 ± 1.8	22.2 ± 1.5	3.0 × 10^−4^	4.4 × 10^−4^	2.1 × 10^−3^
Intermediate frontal sulcus	L	Depth	17.1 ± 2.1	14.9 ± 1.5	3.5 × 10^−5^	2.6 × 10^−2^	3.2 × 10^−2^
Calcarine fissure	L	Depth	31.4 ± 5.9	24.9 ± 6.4	1.5 × 10^−4^	1.1 × 10^−3^	1.2 × 10^−2^
Subparietal sulcus	R	Depth	14.3 ± 2.9	11.4 ± 2.8	2.0 × 10^−4^	5.6 × 10^−3^	5.5 × 10^−3^
Superior temporal sulcus	L	Depth	24.7 ± 2.4	22.7 ± 4.0	7.3 × 10^−4^	1.6 × 10^−3^	2.6 × 10^−2^
Sylvian (lateral) fissure	L	Length	282.7 ± 42.3	335.9 ± 58.1	3.2 × 10^−4^	8.9 × 10^−4^	1.4 × 10^−3^

⁎ Same analyses carried out adding sex and handedness as two additional covariates of no interest.

⁎⁎ Same analyses carried out on the residuals obtained after partialling out the effect of intracranial volume (ICV).

### 3.1 Results consistent with voxel-based findings of cortical atrophy

**Fig. 2:**
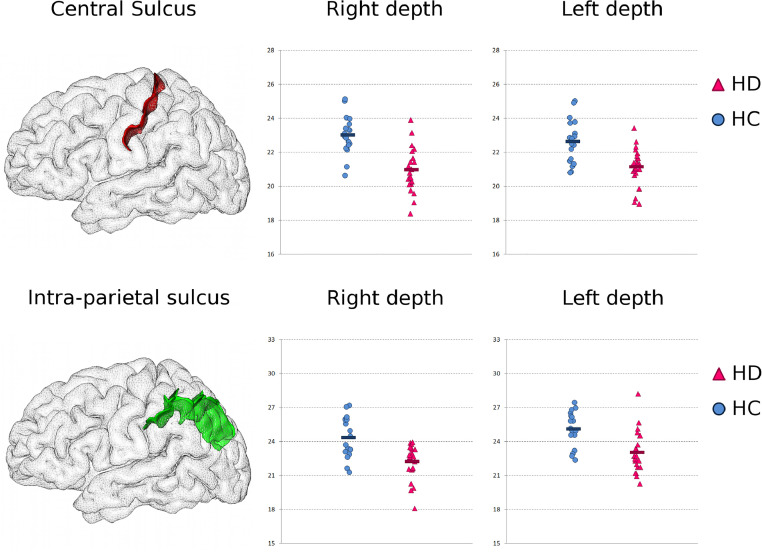
**Bilateral results consistent with cortical atrophy in HD: shallower central and intra-parietal sulcus in HD.***T**op*****: Central Sulcus.***Left*, 3D rendering of the left central sulcus in one healthy subject. *Middle and Right*, maximal depth of the right and left central sulcus in the healthy controls (HC, *n* = 18, in blue circles, average in dark blue), and in the HD participants (*n* = 23, in magenta triangles, average in dark magenta) (a.u.). ***Bottom*****: Intra-Parietal Sulcus.** Same representation as above.

In line with the literature and our previous voxel-based results based on the same HD population (18 out of 23 HD participants in common) (Douaud et al., 2006), we found the strongest difference in the left central sulcus, with a significant reduction of depth of more than 8.8% in the HD patients (see Table 2, Figs. 1 and 2). The right central sulcus depth was also found significantly reduced in HD (−6.6%). The other sulcus significantly different bilaterally in the patients compared with the healthy controls was the intra-parietal sulcus, which was shallower on the left by 8.3%, and on the right by 8.7% (Table 2, Figs. 1 and 2).

### 3.2 Further cortical atrophy findings

**Fig. 3:**
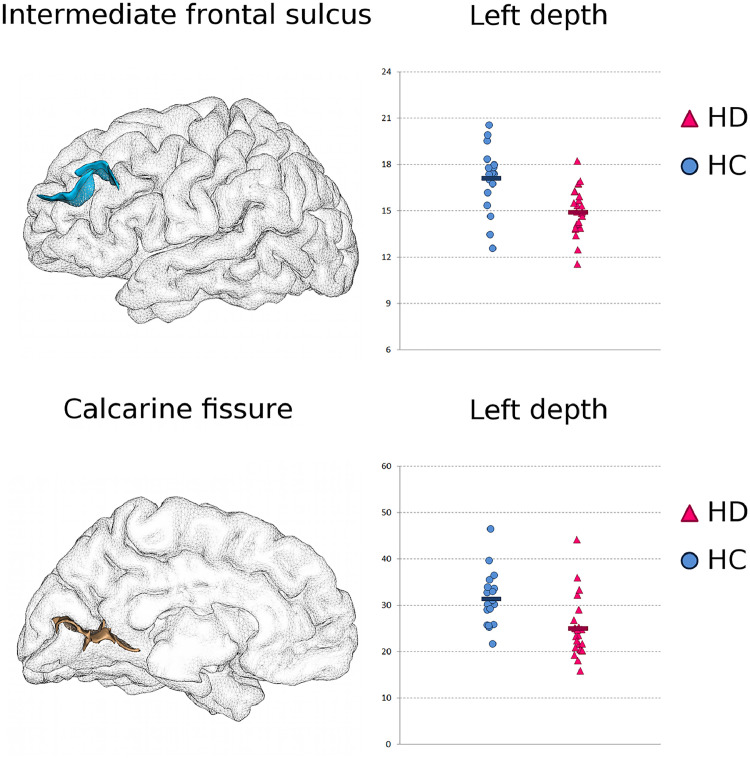
**Additional left-lateralised results consistent with cortical atrophy in HD: shallower intermediate frontal sulcus and calcarine fissure.*****Top*****: Intermediate Frontal Sulcus.***Left*, 3D rendering of the left intermediate frontal sulcus in one healthy subject. *Right*, maximal depth of the left intermediate frontal sulcus in the healthy controls (HC), and in the HD patients (a.u.). ***Bottom*****: Calcarine Fissure.** Same representation as above.

This individual measure approach further revealed significantly shallower left intermediate frontal sulcus, decreased by 12.9% in the patients, in line with the consistent cortical post mortem observation of dorso-lateral prefrontal cortex atrophy (Table 2, Figs. 1 and 3). The depth of right subparietal sulcus (in the precuneus) and left superior temporal sulcus were also found significantly decreased in the patients (Table 2). Remarkably, we also found a strong decrease in depth of the left calcarine fissure of 20.6% in the HD patients, despite this cortical area not projecting onto the basal ganglia (Table 2, Figs. 1 and 3).

### 3.3 Evidence for abnormality of neurodevelopment in HD

**Fig. 4:**
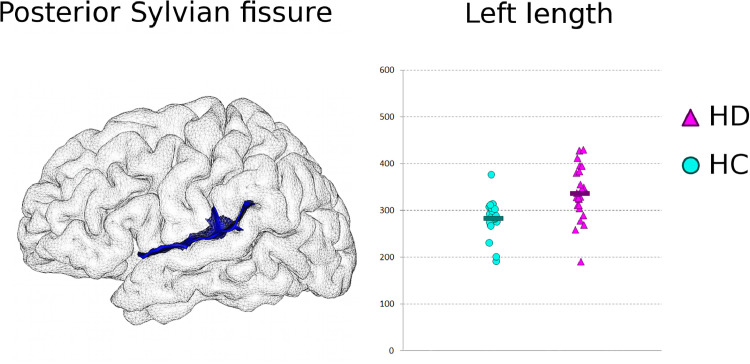
**Evidence for abnormality of neurodevelopment in HD: longer left posterior Sylvian (lateral) fissure in HD.***Left*, 3D rendering of the left posterior Sylvian fissure in one healthy subject. *Right*, length of the left posterior Sylvian fissure in the healthy controls (HC, *n* = 18, turquoise circles), and in the HD participants (*n* = 23, mauve triangles) (a.u.).

Beyond these consistent findings of reduced sulcal depth pointing at cortical degeneration, the sulcal analysis also revealed a strong difference in the length of the posterior Sylvian fissure, with a length increased by 18.9% for the HD participants compared with healthy controls (Table 2, Figs. 1 and 4). This measure of the sulcal length, on the contrary to that of sulcal depth which is a probably marker of colocalised atrophy, is more likely the hallmark of an altered developmental process during the formation of the sulcal roots (Auzias et al., 2014; Cachia et al., 2014; Muellner et al., 2015).

**Fig. 5:**
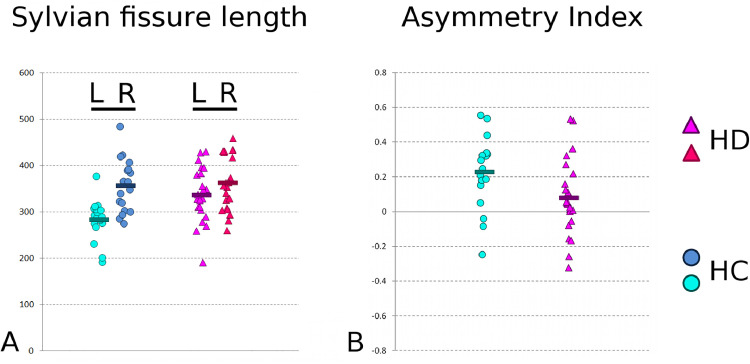
**Evidence for abnormality of neurodevelopment in HD: absence of asymmetry in the posterior Sylvian fissure in HD. A.** There is a natural asymmetry between left and right length of the posterior Sylvian fissure in healthy controls (HC) (a.u.). In HC, the left fissure is shorter on average by almost 20%. By contrast, there is almost no difference on average in the HD patients. For the patients, the left Sylvian fissure is only shorter by less than 6% on average. **B.**This absence of asymmetry is also found at the single-subject level: the asymmetry index of the Sylvian fissure length is close to 0 in the HD patients.

Investigating further the measure of length in the Sylvian fissure, it is clear that, in healthy controls, this fissure is in fact considerably shorter in the left hemisphere than the right (Fig. 5). Rather than simply seeing the finding in the left Sylvian fissure as a mere *longer* sulcus in the patients, it can thus be interpreted more appropriately as an almost complete *absence of asymmetry* for this sulcus in HD, asymmetry that is normally found in healthy participants (Fig. 5). The left Sylvian fissure is indeed shorter than the right by 18.8% in the healthy participants, compared with only 5.5% in HD. This absence of asymmetry is further maintained at the single-subject level, as the asymmetry index between left and right Sylvian fissure length, AI=(R-L)/0.5*(*R* + *L*), is significantly decreased (towards 0) in the HD group (*P* = 0.02, *n* = 41).

As the length of the left posterior Sylvian fissure seemed to be a hallmark of abnormal asymmetry in HD, we further investigated within this group if it was associated with their striatal volumetric asymmetry, as measured using careful manual segmentation of the subcortical structures (Douaud et al., 2006). We found that it was significantly correlated with such subcortical asymmetry (r_23_=0.49, 24% of variance explained, *P* = 0.017, **Supplementary Figure 1**).

Finally, we established that the abnormal length of the Sylvian fissure in HD was not correlated with either disease burden (r_21_ = 0.08, *P* = 0.38) or TFC (r_20_ = 0.19, *P* = 0.21).

### 3.4 Post-hoc correlations with clinical scores

We first reduced the set of scores to those that did not share more than 50% of explained variance (*r* > 0.70). This allowed us to assess correlations between the significant sulcal findings of depth and length with the Motor UHDRS, Behavioural UHDRS, Stroop Interference (highly correlated with Stroop Word and Colour, and Digit Symbol), Functional Assessment (highly correlated with Independence Scale), TFC and Sum Fluency (where we summed the two runs, and which was highly correlated with the MATTIS). Associations are summarised in Supplementary Table 1, but briefly: these showed an association between Stroop Interference and depth of the right intra-parietal sulcus (r_20_ = 0.40, 16% of variance explained, *P* = 0.04), Functional Assessment and depth of the left intermediate frontal sulcus (r_20_ = =−0.46, 21% of variance explained, *P* = 0.02), and Behavioural UHDRS and depth of the left calcarine fissure (r_20_ = −0.52, 28% of variance explained, *P* = 0.009). After regressing age out, we also found an association between the sum of the fluency scores and the depth of the left intermediate frontal sulcus (**Supplementary Table 1**).

## 4 Discussion

This is the first study of sulcal morphology carried out in HD. The motivation for this study was two-fold. First, it was prompted by a string of published evidence that has established early cortical degeneration in HD, whether in the same early HD population (18 out of 23 in common): (Douaud et al., 2006), or even in premanifest HD: (Thieben et al., 2002; Rosas et al., 2005). A previous global morphological study found a global decrease of sulcal depth in HD (Nopoulos et al., 2007). Here, our results might explain this global effect by showing a clear, localised decrease in depth of the central and intra-parietal sulcus in both hemispheres, right sub-parietal sulcus, and left intermediate frontal sulcus, calcarine fissure and superior temporal sulcus. Second, as this sulcal morphometry approach may be able to differentiate underlying degenerative and developmental processes, it was further motivated by two recent *in vivo* studies in gene carriers showing the first signs of abnormal development using anthropometric measurements (Nopoulos et al., 2011; Lee et al., 2012). Remarkably, our sulcal analysis revealed a substantial difference in the imprint of the posterior Sylvian fissure, namely an absence of asymmetry in the HD population between left and right hemispheres, suggesting a very early insult to the developing neocortex.

A shallower central sulcus in our HD participants can be easily related to the most consistent loss of cortical grey matter in the precentral and postcentral gyri observed in a meta-analysis in HD (Dogan et al., 2013), not least in the same patients (Douaud et al., 2006). The depth of the intraparietal sulcus was also significantly decreased bilaterally in the HD participants, particularly so in the left hemisphere (Fig. 3). The left intraparietal sulcus is, together with the premotor and primary sensorimotor cortex, the cortical region found to also discriminate best between premanifest and manifest HD in a meta-analysis (Dogan et al., 2013). In addition, the right intraparietal sulcus depth correlated in the patients with the Stroop Interference (r_20_==0.40, **Supplementary Table 1**), a measure of selective attention whose functional network is centred on the intra-parietal sulcus (Hedden et al., 2012). When we also investigated, as an additional analysis, the surface measure of the sulci, we found that the strongest differences were found bilaterally in the intraparietal sulcus (**Supplementary Table 2**). While mainly redundant (and less sensitive) than the measure of sulcal depth, the surface of sulci solely revealed a significant difference in the left olfactory sulcus, which might be linked to the smell deficits consistently observed in HD (Paulsen et al., 2017).

Findings of a left-lateralised degeneration around the intermediate frontal sulcus concur with the wealth of post-mortem evidence on the injury to the dorso-lateral prefrontal cortex e.g., (Hedreen et al., 1991; Halliday et al., 1998). As it was not detected using VBM (Douaud et al., 2006), this suggests that the method used here might be sensitive to detect very early signs of prefrontal degeneration, which are typically seen at later stages of HD (Rosas et al., 2008). For instance, total functional capacity score (TFC) ranging from 1 to 13 was found to correlate with left prefrontal areas (Rosas et al., 2008). In our predominantly stage I HD population, where TFC range was more limited, we found similar associations specifically between the depth of the intermediate frontal sulcus and the UHDRS measures o Functional Assessment (r_20_ = =−0.46), and at a trend level with TFC (r_20_ = 0.31)(**Supplementary Table 1**).

Decrease in depth of the left calcarine fissure could seem surprising at first, as this part of the brain is not connected to the striatum. But it is in fact a result consistent with *in vivo* surface-based studies of HD, where degeneration was found in the occipital lobe and in particular around the left calcarine fissure (Rosas et al., 2002; Rosas et al., 2008), as well as with *post mortem* studies (Halliday et al., 1998). Indeed, while cortical degeneration in HD had been initially thought to be a secondary event due to the striatal degeneration, it is more likely that both primary and secondary degenerative processes co-exist in the cortex (Rub et al., 2015). This is further supported by histopathological findings showing damage to layer VI of the cortex that does not project to the striatum (Hedreen et al., 1991). Of note, the decrease in depth in the calcarine fissure is the strongest in terms of effect size (more than 20%) compared with all other sulci found shallower in HD. Intriguingly, the depth of the left calcarine fissure was correlated with the behavioural UHDRS score (r_20_ = =−0.52, **Supplementary Table 1**). This association might perhaps be related to the association observed between behavioural symptoms – visual hallucinations and depression – and this specific region of the brain also seen in Parkinson's disease (Matsui et al., 2006; Hu et al., 2015).

Interestingly, this sulcal analysis also revealed an increase of nearly 20% in the length of the left posterior Sylvian fissure in HD compared with the healthy participants. The consistent decrease of depth found in various sulci are consistent with a neurodegenerative process, and thus mainly consistent with volume-based and surface-based findings. A significant difference in the length of one sulcus, on the contrary, is more difficult to be interpreted, especially in light of the absence of colocalised atrophy, and the lack of association with disease stage or burden, and age. As such, it is more likely related to an altered development. This left peri‑Sylvian region is for instance well known to be associated with functional language lateralisation and specialisation, although it did not correlate with verbal fluency (Table 1), the only language-related measure available in our HD population. Morphological anomalies in this brain region have been found in population with neurodevelopmental disorders, such as stuttering and in children with dyslexia (Foundas et al., 2004; Kibby et al., 2004; Cykowski et al., 2008). It is also connected by white matter tracts that are the only fibre bundles showing the effect of genetic associations with handedness (Wiberg et al., 2019). However, as shown in the Results section, healthy development typically leads to a strong asymmetry between the two hemispheres – in fact the strongest asymmetry found across the entire cortex, as demonstrated for instance in preterm newborns (Dubois et al., 2010). Our result in the posterior Sylvian fissure therefore demonstrates *an absence of asymmetry in HD*, compared with normal development. Interestingly, differences in sulcal asymmetry have recently demonstrated to be key in understanding differences in developmental processes (Kloppel et al., 2010; Cachia et al., 2014; Leroy et al., 2015). This altogether suggests that the abnormal length of the left posterior Sylvian fissure in HD might bear the hallmark of an early, altered developmental process. As the formation of the Sylvian fissure appears early *in utero*, and marked asymmetry is specifically found in this region in preterm newborns (Dubois et al., 2010), this likely indicates the foetal timing of a disease-related genetic interplay with neurodevelopment. In our HD population, the length of the left posterior Sylvian fissure was further significantly associated with the striatal volumetric asymmetry, as measured using careful manual segmentation of the subcortical structures (r_23_ = 0.49, 24% of variance explained, *P* = 0.017, **Supplementary Figure 1**) (Douaud et al., 2006). Such striatal asymmetry in turn explains a substantial part of the variance in two fundamental UHDRS measures in our cohort: TFC (r_20_ = =−0.49, 24% of variance explained, *P* = 0.027) and Independence Scale (r_19_ = =−0.59, 35% of variance explained, *P* = 0.0075). It could thus be that the subcortical volume asymmetry seen in the striatum of HD patients is both a combination of developmental and degenerative processes.

Compared with a technique such as VBM, this specific sulcal approach cannot show precisely where some of the abnormalities might be localised along a sulcus (e.g., dorsal vs. ventral part of the central sulcus). Newest developments might be able to resolve these limitations (Coulon et al., 2015). In any case, it revealed in the same HD population (18 out of 23 in common), and using the same statistical model, significant differences in areas where the VBM analysis had failed to detect a loss of volume or morphology: the right precuneus, as well as the left dorso-lateral prefrontal cortex, primary visual cortex, superior temporal cortex and peri‑Sylvian region (Douaud et al., 2006). Other VBM studies, possibly because of larger sample size or more advanced HD population, have in some cases demonstrated voxelwise differences in those cortical regions where only our sulcal approach revealed abnormalities (Muhlau et al., 2007; Scahill et al., 2013; Minkova et al., 2018). The sample size of this study is also limited, but we made sure to only present in the main manuscript sulcal group differences surviving correction for multiple comparisons (as an indication, top 20 results in **Supplementary Table 3**). This relatively small sample size also meant large effect sizes for our significant results, such as a difference of 19% in length of the Sylvian fissure, or of 21% in the depth of the calcarine fissure. Finally, another clear limitation is that our participants were already symptomatic. Especially for the findings in the Sylvian fissure, a study on (ideally young) gene carriers far from the onset of symptoms - such as done in Lee et al. (2012) - should confirm the pre-existing nature of this sulcal abnormality, and in particular of its distinctive asymmetry in HD.

In summary, we used for the first time a detailed analysis of sulcal morphology in HD. This approach, which precisely targets cortical features, offers complementary sources of information, not only to conventional voxel- and vertex-wise approaches, but also in how they relate to different underlying physiopathological processes, and could help detect subtle neurodevelopmental abnormalities that would otherwise go unnoticed in other degenerative disorders with a genetic susceptibility. It revealed in HD abnormalities consistent with a neurodegenerative process, but also importantly with an altered neurodevelopment. While the atrophy found in the left visual cortex adds to the increasing wealth of data indicative of a primary cortical degeneration in HD, this study provides, to the best of our knowledge, the first *in vivo* indication of an interplay between disease and neocortical development.

## CRediT authorship contribution statement

**Jean-Francois Mangin:** Conceptualization, Formal analysis, Software, Writing - original draft. **Denis Rivière:** Formal analysis, Software, Writing - original draft. **Edouard Duchesnay:** Formal analysis, Software. **Yann Cointepas:** Software. **Véronique Gaura:** Resources. **Christophe Verny:** Resources. **Philippe Damier:** Resources. **Pierre Krystkowiak:** Resources. **Anne-Catherine Bachoud-Lévi:** Resources, Funding acquisition. **Philippe Hantraye:** Supervision, Funding acquisition. **Philippe Remy:** Supervision, Resources, Funding acquisition. **Gwenaëlle Douaud:** Conceptualization, Resources, Investigation, Formal analysis, Funding acquisition, Writing - original draft, Writing - review & editing.

## Declaration of Competing Interest

The authors declare no competing financial interests.

## Appendix Supplementary materials

Image, application 1
