# Supplementary material for: Neocortical morphometry in Huntington's disease: Indication of the coexistence of abnormal neurodevelopmental and neurodegenerative processes
Source: Neuroimage Clin. 2020 Feb 13;26:102211. doi: 10.1016/j.nicl.2020.102211 (PMC7044794; doi:10.1016/j.nicl.2020.102211)
Supplement: Supplementary file 1 [file mmc1.docx]

**Supplementary Table 1: Correlation results between all non-collinear clinical scores and the significant (FDR-corrected) sulcal differences between HD (n=23) and healthy controls (HC, n=18).**

| **Behavioural & Cognitive** | **Sulci** | **Pearson r** | **% variance** | ***P* value** | ***P* value*** |
| --- | --- | --- | --- | --- | --- |
| **Motor UHDRS** | Calcarine fissure, left depth | 0.33 | 11 | 0.080 | 0.083 |
|  | Central sulcus, right depth | -0.32 | 10 | 0.084 | 0.096 |
|  | Sylvian fissure, left length | 0.29 | 8 | 0.106 | 0.105 |
|  | Central sulcus, left depth | -0.29 | 8 | 0.110 | 0.112 |
|  | Intra-parietal sulcus, right depth | -0.27 | 7 | 0.126 | 0.134 |
|  | Superior temporal sulcus, left depth | 0.22 | 5 | 0.171 | 0.158 |
|  | Intra-parietal sulcus, left depth | -0.13 | 2 | 0.295 | 0.241 |
|  | Intermediate frontal sulcus, left depth | -0.11 | 1 | 0.315 | 0.277 |
|  | Subparietal sulcus, right depth | -0.08 | 1 | 0.361 | 0.308 |
| **Stroop Interference** | **Intra-parietal sulcus, right depth** | **0.40** | **16** | **0.039** | **0.036** |
|  | Intra-parietal sulcus, left depth | -0.21 | 5 | 0.182 | 0.175 |
|  | Intermediate frontal sulcus, left depth | 0.21 | 4 | 0.187 | 0.187 |
|  | Superior temporal sulcus, left depth | 0.19 | 4 | 0.212 | 0.205 |
|  | Sylvian fissure, left length | 0.18 | 3 | 0.224 | 0.215 |
|  | Subparietal sulcus, right depth | 0.17 | 3 | 0.243 | 0.224 |
|  | Central sulcus, right depth | 0.16 | 3 | 0.251 | 0.250 |
|  | Calcarine fissure, left depth | -0.13 | 2 | 0.287 | 0.285 |
|  | Central sulcus, left depth | -0.12 | 1 | 0.311 | 0.312 |
| **Sum Fluency** | **Intermediate frontal sulcus, left depth** | **0.30** | **9** | **0.097** | **0.028** |
|  | Central sulcus, left depth | 0.29 | 8 | 0.107 | 0.113 |
|  | Central sulcus, right depth | 0.16 | 3 | 0.250 | 0.329 |
|  | Superior temporal sulcus, left depth | 0.11 | 1 | 0.315 | 0.331 |
|  | Sylvian fissure, left length | -0.10 | 1 | 0.339 | 0.335 |
|  | Calcarine fissure, left depth | 0.10 | 1 | 0.345 | 0.356 |
|  | Intra-parietal sulcus, right depth | -0.08 | 1 | 0.368 | 0.374 |
|  | Subparietal sulcus, right depth | -0.05 | 0 | 0.414 | 0.432 |
|  | Intra-parietal sulcus, left depth | 0.01 | 0 | 0.480 | 0.461 |
| **Functional Assessment** | **Intermediate frontal sulcus, left depth** | **-0.46** | **21** | **0.021** | **0.049** |
|  | Central sulcus, left depth | -0.35 | 12 | 0.067 | 0.062 |
|  | Superior temporal sulcus, left depth | 0.28 | 8 | 0.116 | 0.142 |
|  | Calcarine fissure, left depth | 0.21 | 4 | 0.192 | 0.178 |
|  | Subparietal sulcus, right depth | -0.13 | 2 | 0.299 | 0.273 |
|  | Sylvian fissure, left length | -0.12 | 1 | 0.308 | 0.304 |
|  | Intra-parietal sulcus, left depth | 0.08 | 1 | 0.376 | 0.326 |
|  | Intra-parietal sulcus, right depth | 0.05 | 0 | 0.414 | 0.423 |
|  | Central sulcus, right depth | -0.04 | 0 | 0.439 | 0.456 |
| **Behavioural UHDRS** | **Calcarine fissure, left depth** | **-0.52** | **28** | **0.009** | **0.010** |
|  | Subparietal sulcus, right depth | -0.37 | 13 | 0.056 | 0.082 |
|  | Superior temporal sulcus, left depth | -0.24 | 6 | 0.156 | 0.133 |
|  | Intra-parietal sulcus, left depth | 0.23 | 5 | 0.170 | 0.144 |
|  | Intra-parietal sulcus, right depth | 0.22 | 5 | 0.175 | 0.196 |
|  | Central sulcus, left depth | -0.13 | 2 | 0.287 | 0.208 |
|  | Central sulcus, right depth | -0.11 | 1 | 0.322 | 0.279 |
|  | Sylvian fissure, left length | -0.08 | 1 | 0.371 | 0.367 |
|  | Intermediate frontal sulcus, left depth | -0.06 | 0 | 0.399 | 0.470 |
| **Total functional capacity** | Intermediate frontal sulcus, left depth | 0.31 | 9 | 0.095 | 0.074 |
|  | Sylvian fissure, left length | 0.19 | 4 | 0.206 | 0.207 |
|  | Superior temporal sulcus, left depth | -0.18 | 3 | 0.221 | 0.218 |
|  | Intra-parietal sulcus, right depth | 0.16 | 3 | 0.251 | 0.251 |
|  | Central sulcus, left depth | 0.15 | 2 | 0.265 | 0.265 |
|  | Calcarine fissure, left depth | -0.12 | 1 | 0.313 | 0.313 |
|  | Subparietal sulcus, right depth | -0.09 | 1 | 0.357 | 0.356 |
|  | Central sulcus, right depth | 0.07 | 1 | 0.379 | 0.377 |
|  | Intra-parietal sulcus, left depth | -0.06 | 0 | 0.406 | 0.407 |

*Same correlation analyses carried out after regressing age out

**Supplementary Table 2: Surface differences between HD (n=23) and healthy controls (HC, n=18).** In black, significant differences (FDR-corrected). In bold: sulci showing significant differences only using surface measure. In grey, the sulci showing significant difference in their depth also show clear trends in their surface, except for the calcarine fissure.

| Sulci | Side | *F*-value | *P*-value |
| --- | --- | --- | --- |
| Intra-parietal sulcus | L | 21.0 | 5.5×10^-5^ |
|  | R | 20.8 | 5.9×10^-5^ |
| **Olfactory sulcus** | **L** | **14.5** | **5.1×10^-4^** |
| **Parieto-occipital fissure** | **L** | **13.5** | **7.4×10^-4^** |
| Superior temporal sulcus | L | 11.4 | 1.7×10^-3^ |
| Central Sulcus | L | 10.6 | 2.4×10^-3^ |
|  | R | 8.2 | 7.0×10^-3^ |
| Subparietal sulcus | R | 8.9 | 5.1×10^-3^ |
| Intermediate frontal sulcus | L | 8.8 | 5.3×10^-3^ |
| Calcarine fissure | L | 1.9 | 1.7×10^-1^ |

**Supplementary Table 3: Top 20 sulcal differences between HD (n=23) and healthy controls (HC, n=18).** In black, significant differences (FDR-corrected, n=9). In grey, sulci showing a trend (n=11).

| Sulci | Side | Feature | *F*-value | *P*-value |
| --- | --- | --- | --- | --- |
| Central sulcus  Intra-parietal sulcus | R | Depth | 28.7 | 4.6×10^-6^ |
|  | L | Depth | 22.8 | 2.9×10^-5^ |
| Intermediate frontal sulcus | L | Depth | 22.3 | 3.5×10^-5^ |
| Calcarine fissure | L | Depth | 17.9 | 1.5×10^-4^ |
| Subparietal sulcus | R | Depth | 17.1 | 2.0×10^-4^ |
| Intra-parietal sulcus  Sylvian fissure | R | Depth | 15.9 | 3.0×10^-4^ |
|  | L | Length | 15.8 | 3.2×10^-4^ |
| Central sulcus | L | Depth | 15.1 | 4.0×10^-4^ |
| Superior temporal sulcus | L | Depth | 13.9 | 7.3×10^-4^ |
| Sylvian fissure | R | Depth | 12.4 | 1.2×10^-3^ |
| Internal parietal sulcus | R | Depth | 12.2 | 1.3×10^-3^ |
| Marginal frontal sulcus | L | Depth | 11.4 | 1.8×10^-3^ |
| Superior frontal sulcus | L | Depth | 11.3 | 1.9×10^-3^ |
| Posterior calloso-marginal fissure | R | Depth | 10.7 | 2.3×10^-3^ |
| Marginal frontal sulcus | R | Depth | 10.6 | 2.4×10^-3^ |
| Marginal precentral sulcus | L | Depth | 9.6 | 3.7×10^-3^ |
| Parieto-occipital fissure | L | Length | 9.4 | 4.1×10^-3^ |
| Retrocentral transwerse ramus of the lateral fissure | R | Depth | 9.3 | 4.4×10^-3^ |
| Orbital sulcus | L | Depth | 9.1 | 4.7×10^-3^ |
| Subparietal sulcus | L | Depth | 8.7 | 5.4×10^-3^ |

Note: We excluded the internal frontal sulcus as it would have required *a priori* manual intervention to ensure the robustness of its identification.


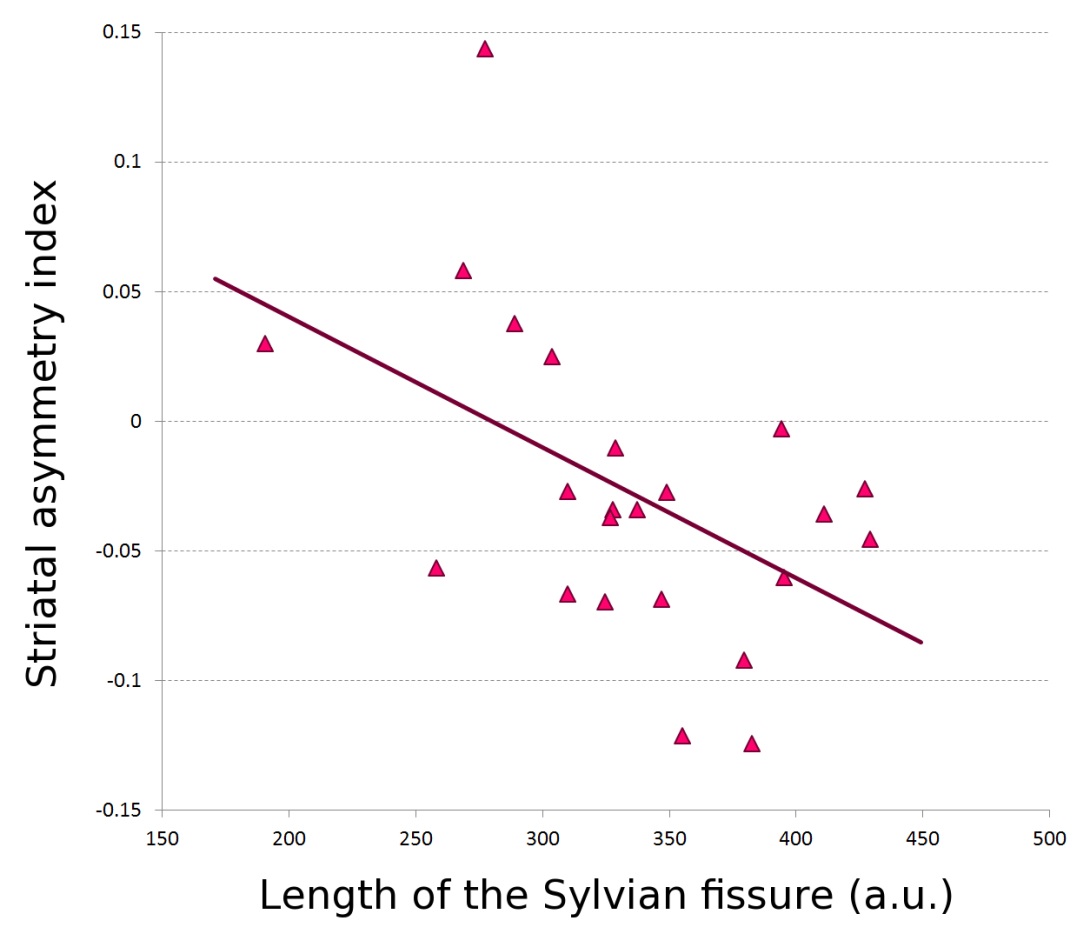


**Supplementary Figure 1: Correlation plot showing the relationship in the HD participants between the abnormal length of the left Sylvian fissure and the asymmetry index in their striatal volumes** (r_23_=0.49, 24% of variance explained, P=0.017)**.**
